# Isolated anterior mediastinal tuberculosis in an immunocompetent patient

**DOI:** 10.1186/s12890-016-0175-7

**Published:** 2016-02-03

**Authors:** S. Maguire, S. H. Chotirmall, V. Parihar, L. Cormican, C. Ryan, C O’Keane, K. Redmond, C. Smyth

**Affiliations:** Department of Gastroenterology, Connolly Hospital, Blanchardstown, Dublin, Dublin 15 Ireland; Lee Kong Chian School of Medicine, Nanyang Technological University, Singapore, Singapore; Department of Respiratory Medicine, Connolly Hospital, Blanchardstown, Dublin, Dublin 15 Ireland; Department of Histopathology, Mater Misericordiae Hospital, Dublin, Dublin 7 Ireland; Department of Cardiothoracic Surgery, Mater Misericordiae Hospital, Dublin, Dublin 7 Ireland

**Keywords:** Tuberculosis, Atypical, Mycobacterium, Mass, Mediastinal

## Abstract

**Background:**

The differential diagnosis of a mediastinal mass is a common challenge in clinical practice, with a wide range of differential diagnosis to be considered. One of the rarer causes is tuberculosis. Atypical presentations of tuberculosis are well documented in immunocompromised patients, but should also be considered in the immunocompetent.

**Case presentation:**

This case outlines a previously healthy 22 year-old immunocompetent male presenting with worsening chest pain, positional dyspnea, dry cough and dysphagia. Chest x-ray showed evidence of an isolated anterior mediastinal mass, which was confirmed on computed tomography. A mediastinoscopy was diagnostic as histology revealed necrotizing granulomatous inflammation and the presence of acid-fast bacilli, indicating mediastinal tuberculosis.

**Conclusion:**

Typically the underlying presentation of mediastinal tuberculosis is mediastinal lymphadenitis. This case was unusual in that we detected an isolated large anterior mediastinal mass accompanied by a relatively small burden of mediastinal lymphadenitis. Cases similar to this have been documented in immunosuppressed patients however in our case no evidence of immunosuppression was found. This case report emphasizes the importance that a detailed and logical pathway of investigation is pursued when encountering a mediastinal mass.

**Electronic supplementary material:**

The online version of this article (doi:10.1186/s12890-016-0175-7) contains supplementary material, which is available to authorized users.

## Background

Mediastinal masses are routinely encountered in clinical practice and a wide range of differential diagnoses must be considered. A less common cause is tuberculosis, a disease recognized for its atypical presentations attributed to its ability to affect almost any organ system. Tuberculosis has an incidence of 126 per 100,000 according to the latest report from the World Health Organization (WHO) [1]. *Mycobacterium tuberculosis* undergoes lympho-heamatogenous dissemination after entering the respiratory tract. Typically the mediastinal and hilar lymph nodes are the first lymphatic tissues *M. tuberculosis* will encounter [2] and it may then spread to other organ systems. Thirty percent of patients present with extra-pulmonary tuberculosis, an example of which is illustrated by this case [3].

## Case presentation

A 22-year-old Nigerian male presented to the accident and emergency department with a 2-week history of worsening chest pain associated with positional dyspnea, dry cough and dysphagia. He had no significant past history, was on no medication and denied any significant family history. The patient was resident in the Republic of Ireland since early childhood and had no recent travel abroad.

**Fig. 1:**
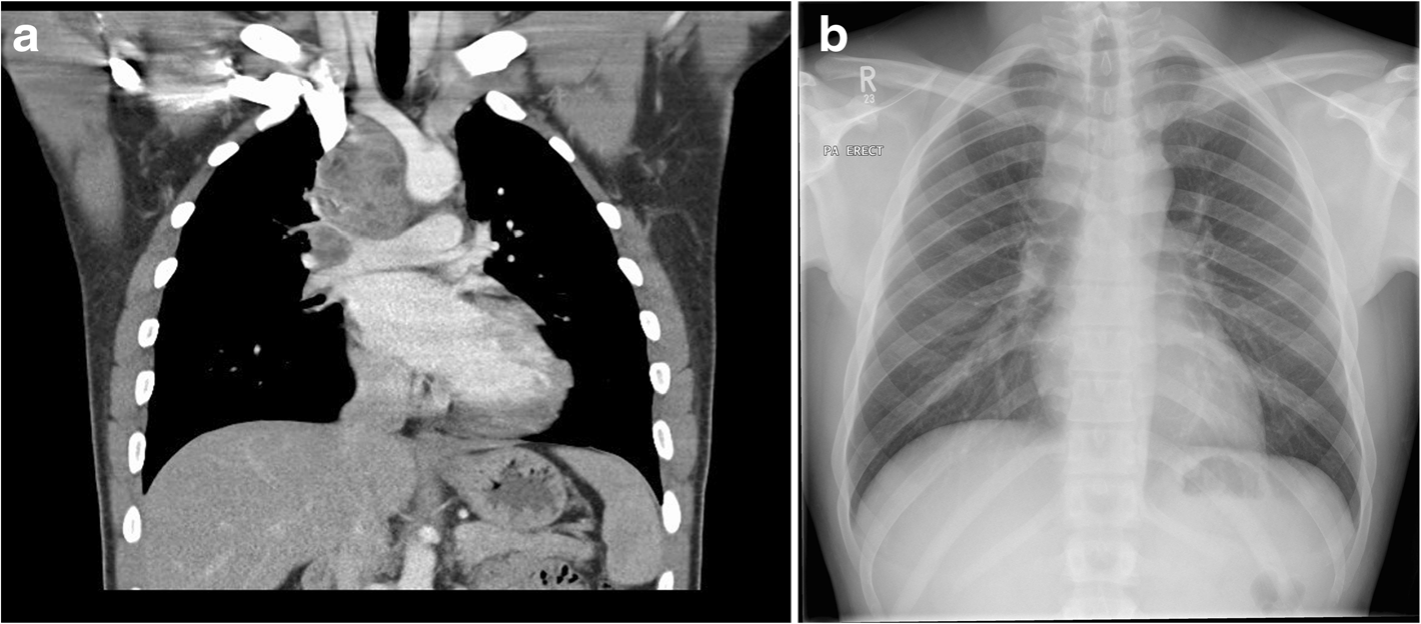
**a** Initial chest x-ray demonstrates soft tissue enlargement within the right paratracheal stripe; **b** Contrast computed tomography of the thorax shows a large right-sided mediastinal mass with accompanying lymphadenopathy encompassing the pre-tracheal, para-tracheal, tracheobronchial, hilar and subcarinal regions. (The images have been placed in reverse order and so the caption does not match the image. The CXR should be Figure 1a and the CT image should be Figure 1b)

Chest radiography illustrated soft tissue enlargement within the right paratracheal stripe (Fig. 1a). Subsequent computed tomography of the thorax with contrast, revealed a large right-sided mediastinal mass with accompanying lymphadenopathy that encompassed the pre-tracheal, para-tracheal, tracheobronchial, hilar and subcarinal regions superior to the right brachiocephalic vein, superior vena cava right pulmonary artery and superior right pulmonary vein (Fig. 1b).

Initial haematological testing revealed the following: C-reactive protein 62 (<5), ESR 102 (<20) and LDH 819 (208–378). Coagulation studies were also abnormal with a PT of 16.3 (11–13.5) and an INR of 1.4. Other laboratory tests including full blood count, liver function test, troponin, electrolytes and glucose were normal as were the tumor markers beta human chorionic gonadotrophin (hCG) and alpha-fetoprotein. Computed tomography of the abdomen and pelvis was also concurrently done and found to be normal, with no evidence of other lymphadenopathy or masses.

Bronchoscopic evaluation illustrated 50 % external compression of the distal trachea with normal distal airways and no endobronchial mass visualized. Trans-bronchial biopsies showed no abnormality and bronchoalveolar lavage (BAL) showed pus cells but no organisms and tested negative for acid-fast bacilli. A full virology screen including HIV, hepatitis B and C were also negative. Whilst lymphoma was our primary working diagnosis due to the patient’s age, mildly elevated LDH and the presence of a mass, tissue confirmation was pursued as initial bronchoscopic biopsies were not confirmatory.

**Fig. 2:**
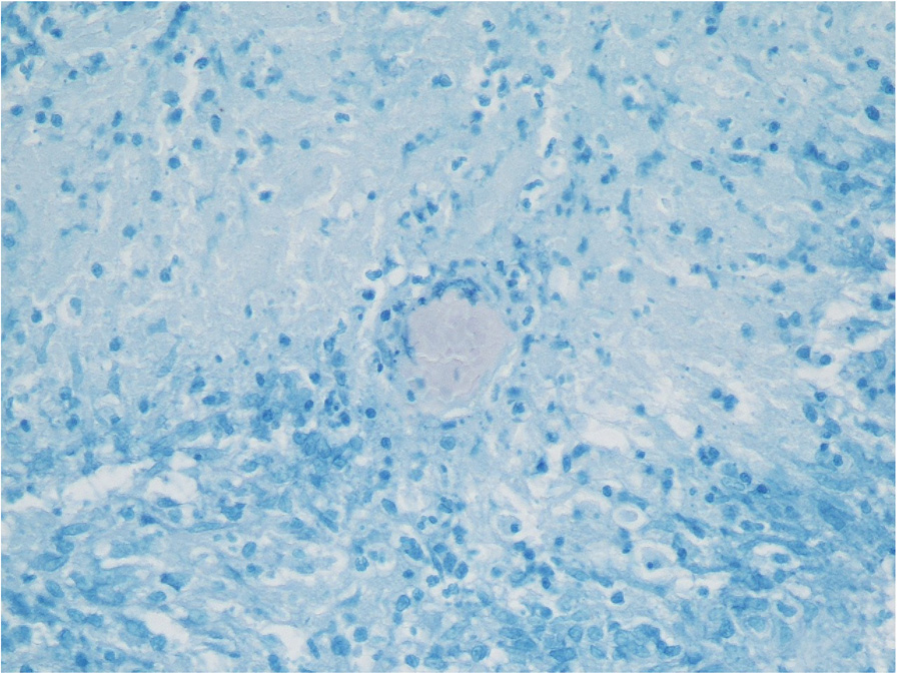
Histology slide of a tissue specimen obtained via mediastinoscopy with Ziehl Neelsen staining shows a single acid-fast bacilli in center of necrosis

**Fig. 3:**
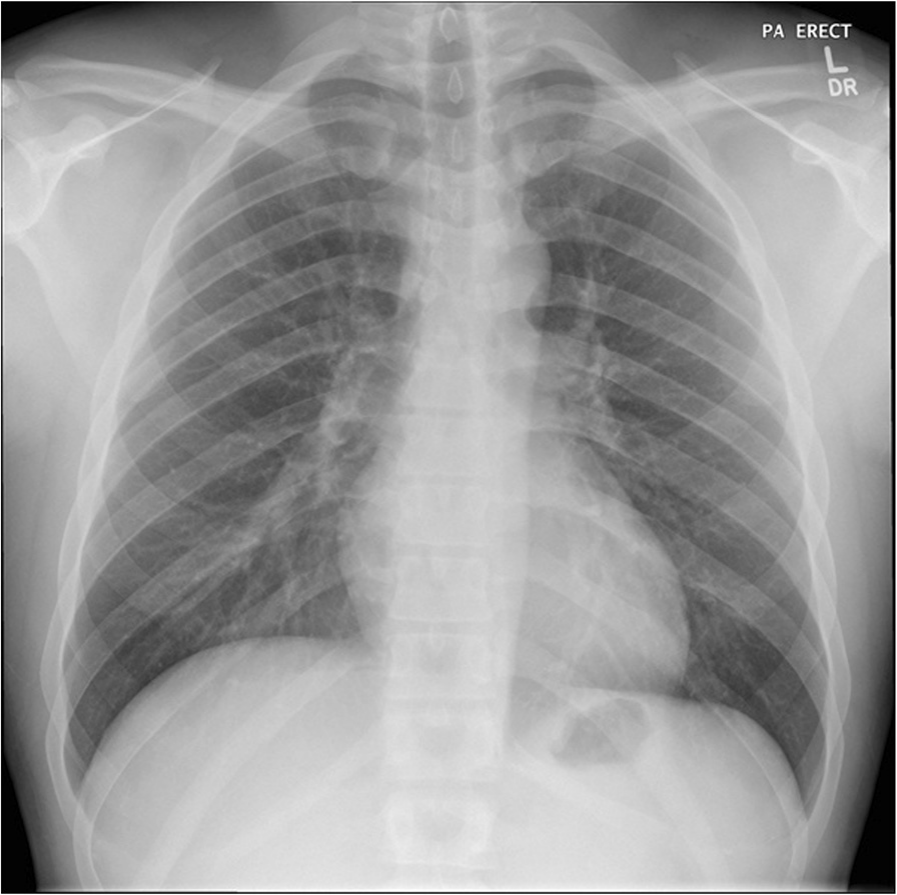
Follow up chest x-ray shows considerable reduction in the soft tissue previously noted in relation to the right paratracheal stripe

A mediastinoscopy was subsequently performed and histology confirmed necrotizing granulomatous inflammation with the presence of acid fast bacilli (AFB) diagnostic of mediastinal tuberculosis (Fig. 2). The patient was commenced on a standard anti-tuberculosis regime for a total of 6 months and has since completed his course of treatment. His most recent chest x-ray (CXR) shows interval improvement (Fig. 3) and he has now been discharged from the respiratory service.

## Conclusions

The differential diagnosis of a mediastinal mass is a common challenge in clinical practice (Table 1)[4]. Patients with these lesions are typically asymptomatic until they develop mass effects, such as chest pain, dyspnea or cough due to compression of local structures. Such symptoms prompted this patient’s presentation. Investigation of such lesions must consider the wide range of differentials. As part of the initial evaluation, a detailed history and physical examination should be performed, looking specifically for lymphadenopathy, thyroid enlargement, testicular masses or evidence of immunosuppression. None of these features were present in this patient. A full haematological screen should be performed including relevant tumor markers including beta hCG and alpha-fetoprotein. Gene Xpert analysis is another well-recognized modality in rapid diagnosis of tuberculosis and should be considered in the work up of a mediastinal mass. Contrast computed tomography (CT) of the thorax is useful to further characterize a mediastinal mass. Radiographic findings suggestive of tuberculosis are a ghost like ring enhancement within a matted mass of nodes [5]. Importantly, tuberculosis has been shown to mimic malignancy both radiologically and clinically [6] hence diagnostic confirmation by histology is imperative prior to therapeutic intervention. This can be obtained by bronchoscopy or endoscopic bronchial ultrasound allowing for fine needle aspirate cytology or transbronchial needle aspirate to obtain a diagnostic pathological specimen.

**Table 1:** Differential diagnosis of a mediastinal mass

Lymphoma	
Thymoma	
Germ cell tumour	
Thyroid enlargement	
Vascular lesion	
Lymphadenopathy	
Cystic lesion (pleuropericardial or bronchogenic)	
Tuberculosis	

There are several and varied presentations of mediastinal tuberculosis including lymph node calcification, oesophagomediastinal fistula, pericardial TB and fibrosing mediastinitis. The underlying cause of these lesions is mediastinal lymphadenitis, which extends to involve surrounding structures creating strictures and fistulation [7]. This case was unusual in that we detected an isolated large anterior mediastinal mass accompanied by a relatively small burden of mediastinal lymphadenitis. Prior cases similar to this have been noted to occur in immunosuppressed individuals such as those with seropositive HIV with a CD4 T lymphocyte count <200/mm^3 but are even more rarely found in HIV positive patients with a CD4 T lymphocyte count >200/mm^3 [8, 9]. Such a presentation is unusual in immunocompetent patients, although has been shown to occur more frequently in patients from countries where TB is endemic [10].

There are few documented cases of tuberculosis presenting as an isolated anterior mediastinal mass in an immunocompetent patient. Comprehensive review of the literature reveals ten similarly described cases with the majority occurring in the pediatric population aged 2 years or younger. Of the published cases on adults all three patients were male with age ranging from 32 to 76 years [11–14]. The masses varied in composition and behavior, with one case describing a cystic mass and another a mass infiltrating the right atrium. Similar to this case all three patients were treated with medical therapy. The limited published data on adult presentations of mediastinal tuberculosis in immunocompetent patients does reveal that presentation is usually associated with mass effect symptoms and that a diagnosis was only revealed following histological confirmation, both features observed in this case [15–20].

In summary, while the aetiology of a mediastinal mass can be difficult to establish because of the wide differential, it is imperative that a through and logical scheme of investigation including tissue diagnosis is pursued. This case aims to highlight the importance of considering tuberculosis in the differential even in immunocompetent patients presenting with an isolated mediastinal mass that cannot otherwise be explained.

### Ethics, consent & permissions

Written informed consent was obtained from the patient for publication of this case report and accompanying images. A copy of the written consent is available for review by the Editor of this journal. This project was carried out within an appropriate ethical framework. This manuscript conforms with the CARE guidelines published in 2013 (see Additional file 1).

### Consent to publish

Written informed consent was obtained from the patient for publication of this case report and accompanying images. A copy of the written consent is available for review by the Editor of this journal.

## Availability of data

N/A.

## Additional file

Additional file 1:
**CARE Checklist (2013) of information to include when writing a case report.** (DOCX 1644 kb)

## Abbreviations

HIV: Human innunodeficiency virus
ESR: Erythrocyte sedimentation rate
LDH: Lactate dehydrogenase
INR: International normalized ratio
PT: Prothrombin time
Beta hCG: Human chorionic gonadotrophin
CXR: Chest x-ray
ZN: Ziehl Neelsen
AFB: Acid fast bacilli
CT: Computed tomography
